# Treatment of Obesity

**Published:** 1889-04

**Authors:** 


### TREATMENT OF OBESITY.

The following, are the objects aimed at in the treatment of obesity.

1. To improve the muscular tone of the heart.
2. To maintain the normal composition of the blood.
3. To regulate the quantity of food in the body.
4. To prevent the deposit of fat.

These objects are obtained by the following means ;

1. The muscle of the heart is strengthened by enforced exercise, such as *climbing heights.* This requires great care, and the exercises must be *graded,* the amount of work being increased as the patient can bear it.

2. To preserve the normal composition of the blood, the food should be chiefly albuminous. It may consist of the lean of roast or boiled beef, veal, mutton, game and eggs. Green vegetables (as cabbage or spinach) may be taken ; fat and carbohydrates only in very limited quantities ; from four to six ounces of bread per diem.

3. To regulate the quantity of fluid in the body, the amount of fluid drunk daily must be limited. One cup (rather less than six ounces) of coffee, tea, or milk morning and evening, and about twelve ounces of wine, and from eight to sixteen ounces of water, should comprise all the fluid consumed in twenty-four hours. Beer is entirely forbidden. The discharge of fluid from the body is promoted by active exercise, and occasionally by a course of baths with packing.

3. To prevent the deposit of fat, the principles of diet already set forth must be carried out as follows :

*Morning.*—One cup of tea or coffee, with a little milk, altogether about six ounces ; bread about three ounces.

*Noon.*—Three to four ounces of soup, seven to eight ounces of roast or boiled beef, veal, game, salad, or a lighter vegetable, a little fish (cooked without fat) if desired, one ounce of bread or farinaceous pudding (never more than three ounces), three to six ounces of fruit, fresh preferred, for dessert. It is desirable at this meal to avoid taking fluids, but in hot weather, or in the absence of fruits, six to eight ounces of light wine may be taken.

*Afternoon.*—The same amount of coffee or tea as in the morning, with, at most, six ounces of water, an ounce of bread as an exceptional indulgence.

*Evening.*—One or two soft boiled eggs, an ounce of bread, perhaps a small slice of cheese. Salad and fruit, six to eight ounces of wine, with four to five ounces of water.

Such briefly summarized, are the principles of this peculiarly German anti-fat regimen, for which, as Dr. Yeo rightly affirms, the credit almost entirely belongs to Professor Oertel, the main features of it having for years been set forth in his writings. These principles seem to commend themselves as sound, though their practical application will require considerable modification to adapt them to the stage of the affection and to the constitution and habits of the patient.—Ex. from *Boston Medical and Surgical Journal.*

